# Supplementary material for: Motor learning in real-world pool billiards
Source: Sci Rep. 2020 Nov 18;10:20046. doi: 10.1038/s41598-020-76805-9 (PMC7674448; doi:10.1038/s41598-020-76805-9)
Supplement: Supplementary file 1 — Supplementary Information. [file 41598_2020_76805_MOESM1_ESM.pdf]

Motor learning in real-world pool billiards

Shlomi Haar, Camille M. van Assel & A. Aldo Faisal

Supplementary Information

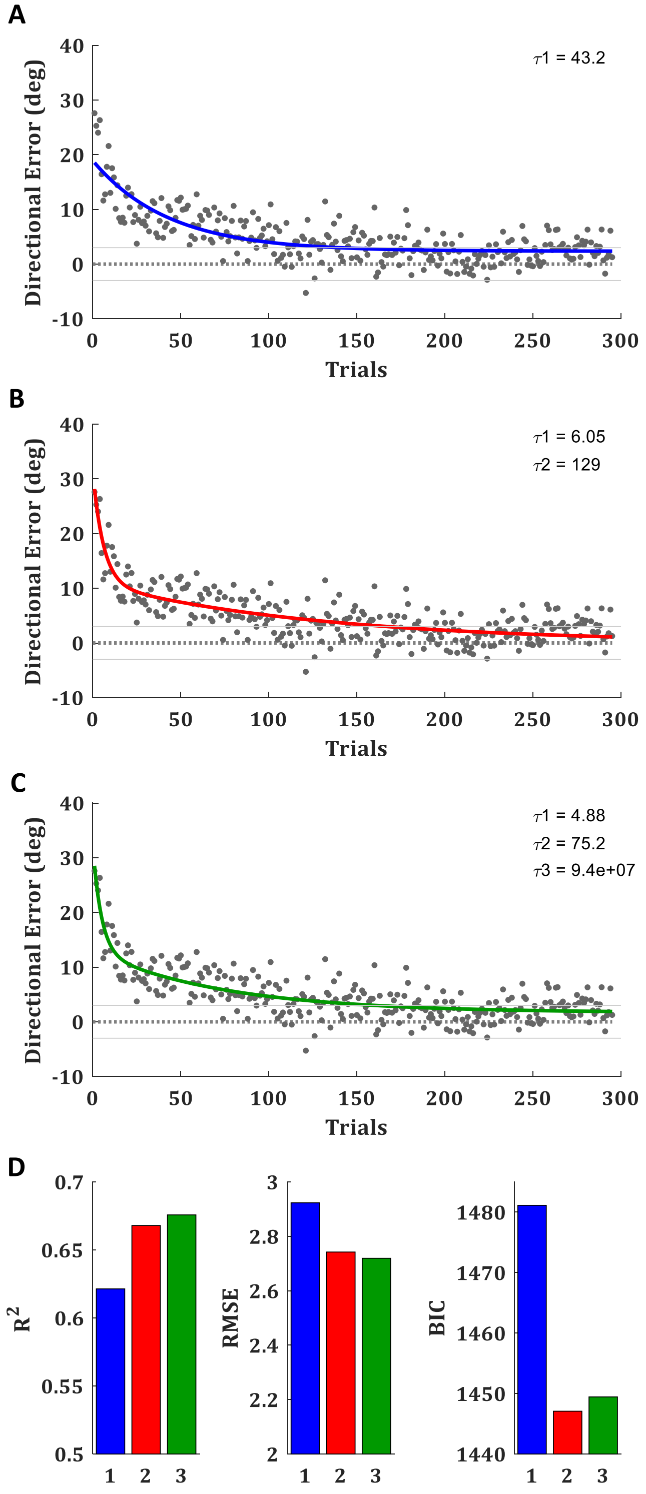

Supplementary Figure 1. *Exponential fits*. (**A-C**) The trial-by-trial directional error of the target-ball (relative to the direction from its origin to the centre of the target pocket), averaged across all subjects, with (**A**) single, (**B**) double, and (**C**) triple exponential fit (blue, red, and green curves, respectively). Grey lines mark the range of successful trials (less than 3 degrees form the centre of the pocket). (**D**) The R square (R^2), root mean square error (SME), and Bayesian information criterion (BIC) for the 3 fits.

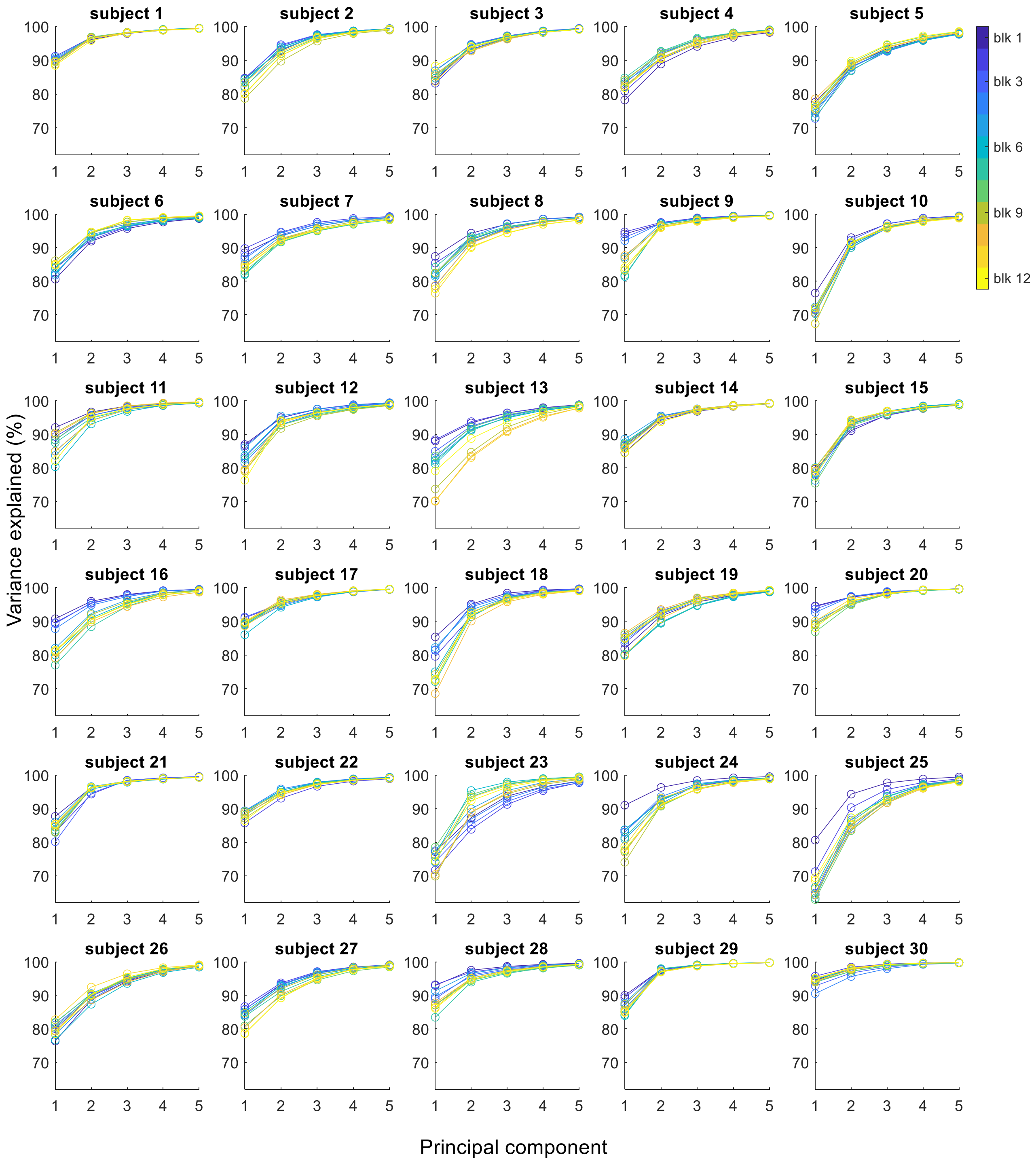

Supplementary Figure 2. *PCA variance explained*. The variance explained by the first five principal components for each subject in each block of trials. Lines are averaged over all trials within each block. Colour-code is by blocks order, from blue in the first block (blk 1) to yellow in the last block (blk 12).

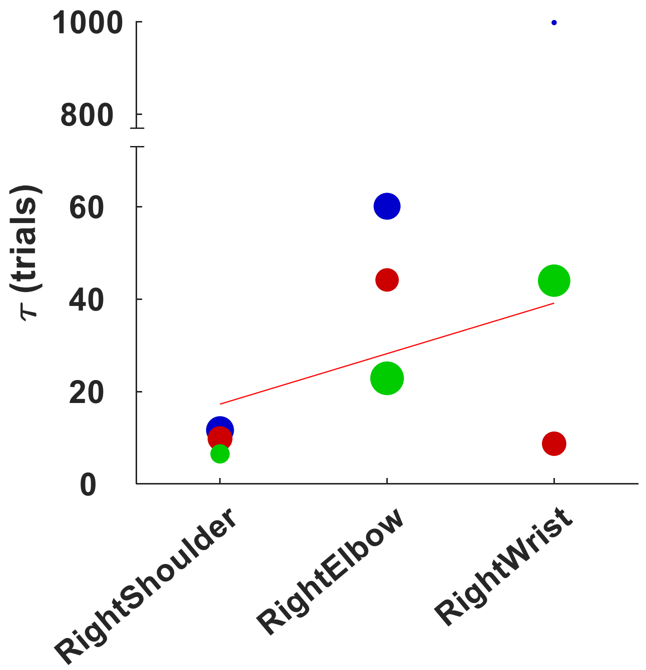

Supplementary Figure 3. *Proximal-to-distal gradient in the VPE over the right arm joints*. The time constants of the exponential fits of the trial-by-trial Velocity Profile Error for all 3 DoF of the right arm joints (from figure 4A). The size of the dot represents the learning rate (B in Supplementary Table 1) and the colour code is the same as in figure 4 (blue: flexion/extension; red: abduction/adduction; green: internal/external rotation). The ret line is weighted linear fit, where the wight is the learning rate.

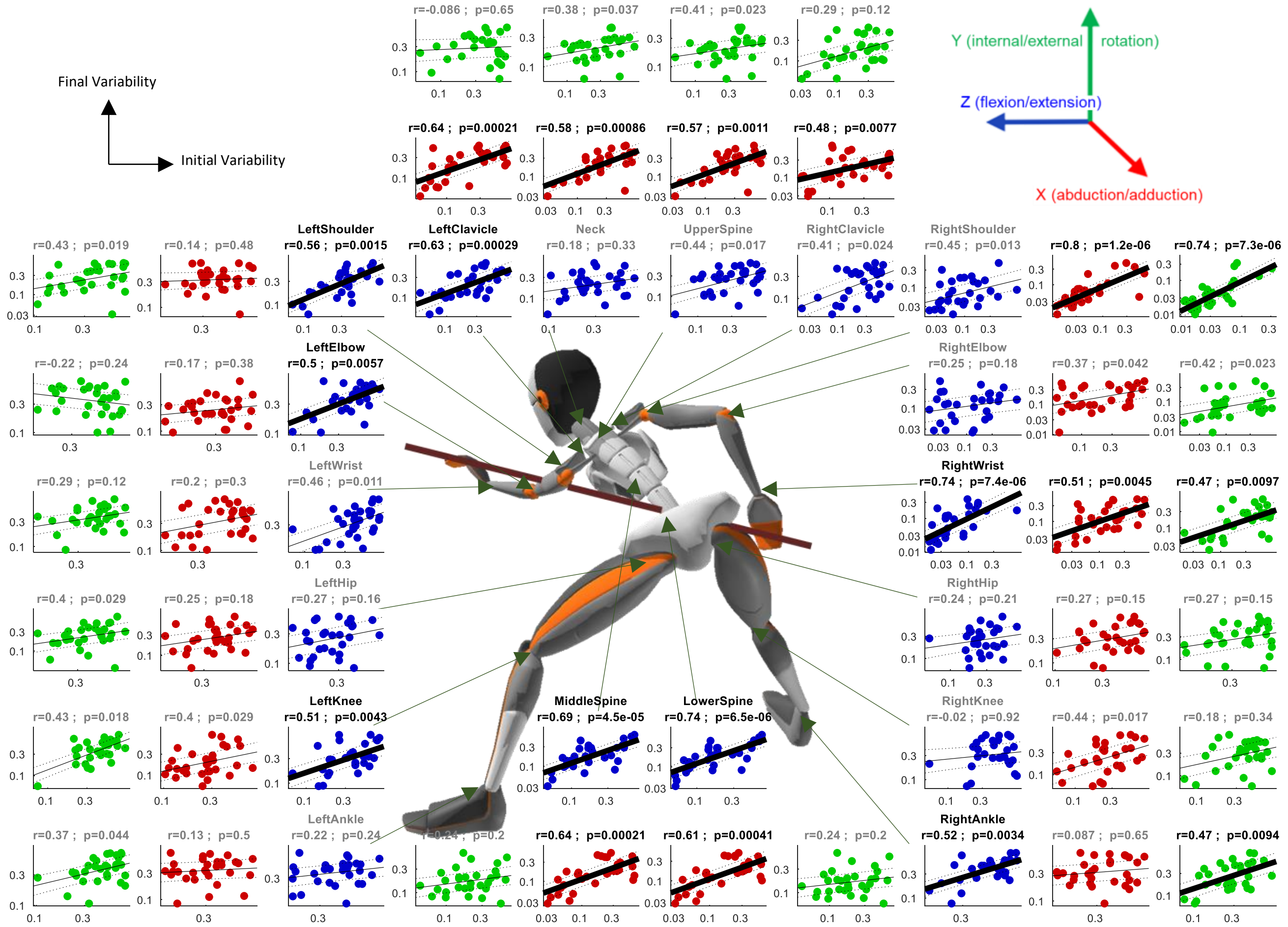

Supplementary Figure 4. *Correlation between subjects' VPE variability over the first block and over the learning plateau*. Presented for all joints in 3 degrees of freedom (DoF) for each joint (blue: flexion/extension, red: abduction/adduction; green: internal/external rotation). Subjects' VPE variability is in logarithmic scale. Correlation values are Spearman rank correlation, regression lines (black) are linear fits with 95% confidence intervals (doted lines).

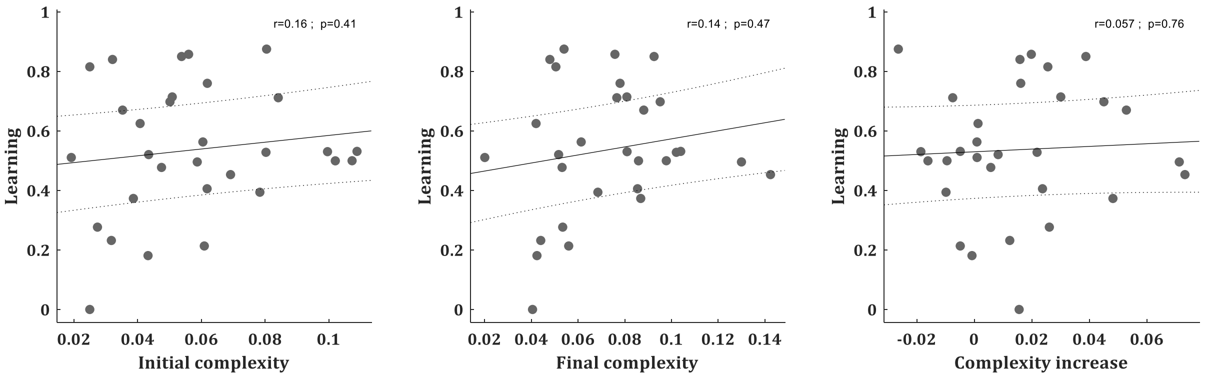

Supplementary Figure 5. *Manipulative complexity and learning across subjects*. (**A**) Correlation between subjects' manipulative complexity over the first block (trials 1-25) and their learning rates. (**B**) Correlation between subjects' manipulative complexity over the learning plateau (trials 201-300) and their learning rates. (**C**) Correlation between subjects' increase in manipulative complexity (from the first block to the learning plateau) and their learning rates. (**A-C**) Correlation values are Spearman rank correlation, regression lines (black) are linear fits with 95% confidence intervals (doted lines).

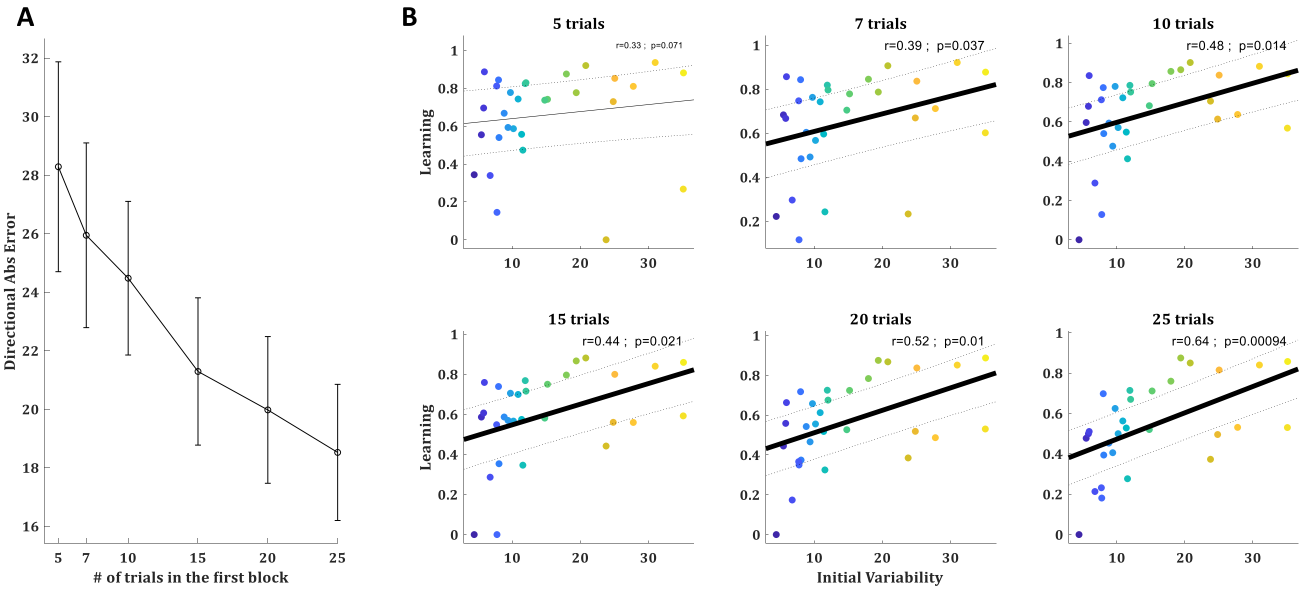

Supplementary Figure 6. *Variability and learning across subjects*. (**A**) The mean absolute directional error of the target-ball over the initial 5,7,10,15,20, and 25 trials, averaged across all subjects. Error bars represent SEM. (**B**) Correlation between subjects' directional variability over the first block (corrected for learning trend, see text) and their learning calculated based on their errors in the first 5,7,10,15,20, or 25 trials respectively in the different panels. Correlation values are Spearman rank correlation, p-values are FDR corrected for multiple comparisons, regression lines (black) are linear fits with 95% confidence intervals (doted lines).

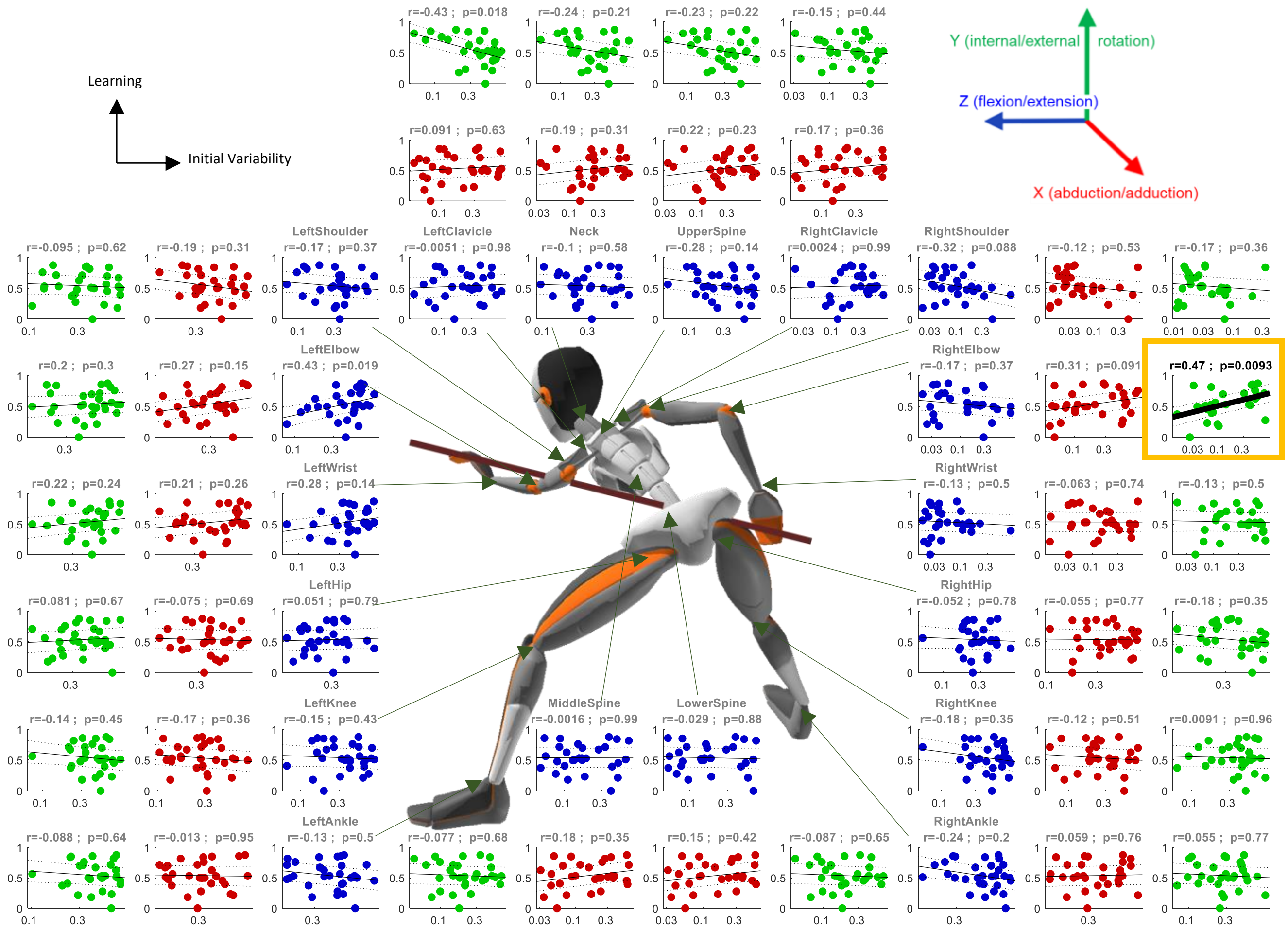

Supplementary Figure 7. *Correlation between subjects' VPE variability over first block and their learning*. Presented for all joints in 3 degrees of freedom (DoF) for each joint (blue: flexion/extension, red: abduction/adduction; green: internal/external rotation). Subjects' VPE variability is in logarithmic scale. Correlation values are Spearman rank correlation, regression lines (black) are linear fits with 95% confidence intervals (doted lines).

Supplementary Table 1. *Exponential fit parameters and Goodness of fit for the VPE (figure 4A)*

jointsLabels	tau	B	R^2	RMSE
LowerSpine flexion	12.16	0.062	0.37	0.011
LowerSpine abduction	211.47	0.037	0.28	0.013
LowerSpine rotation	12.08	0.067	0.38	0.011
MiddleSpine flexion	11.58	0.057	0.33	0.011
MiddleSpine abduction	214.08	0.038	0.28	0.013
MiddleSpine rotation	10.86	0.075	0.39	0.012
UpperSpine flexion	16.92	0.100	0.55	0.014
UpperSpine abduction	15.74	0.099	0.56	0.013
UpperSpine rotation	33.80	0.094	0.64	0.015
Neck flexion	45.07	0.065	0.54	0.014
Neck abduction	15.45	0.099	0.52	0.014
Neck rotation	26.77	0.108	0.68	0.014
RightClavicle flexion	55.13	0.045	0.44	0.012
RightClavicle abduction	27.34	0.073	0.57	0.012
RightClavicle rotation	12.77	0.053	0.30	0.011
RightShoulder flexion	11.64	0.067	0.52	0.008
RightShoulder abduction	9.81	0.053	0.39	0.008
RightShoulder rotation	6.51	0.031	0.19	0.006
RightElbow flexion	60.08	0.063	0.59	0.013
RightElbow abduction	44.14	0.047	0.46	0.012
RightElbow rotation	22.86	0.099	0.72	0.011
RightWrist flexion	998.23	0.000	0.00	0.009
RightWrist abduction	8.71	0.051	0.24	0.010
RightWrist rotation	43.99	0.092	0.71	0.013
LeftClavicle flexion	34.92	0.061	0.46	0.014
LeftClavicle abduction	52.78	0.034	0.26	0.013
LeftClavicle rotation	50.67	0.057	0.44	0.015
LeftShoulder flexion	31.95	0.061	0.48	0.013
LeftShoulder abduction	21.34	0.073	0.45	0.014
LeftShoulder rotation	19.99	0.087	0.50	0.015
LeftElbow flexion	23.16	0.086	0.46	0.017
LeftElbow abduction	43.51	0.072	0.58	0.014
LeftElbow rotation	35.83	0.088	0.50	0.019
LeftWrist flexion	40.01	0.086	0.56	0.017
LeftWrist abduction	30.07	0.111	0.63	0.017
LeftWrist rotation	12.93	0.098	0.36	0.018
RightHip flexion	14.19	0.062	0.39	0.011
RightHip abduction	54.49	0.063	0.50	0.015
RightHip rotation	19.83	0.097	0.55	0.015
RightKnee flexion	48.79	0.112	0.67	0.018
RightKnee abduction	25.79	0.046	0.33	0.012
RightKnee rotation	39.95	0.096	0.65	0.015
RightAnkle flexion	40.10	0.114	0.72	0.016
RightAnkle abduction	28.85	0.051	0.35	0.014
RightAnkle rotation	25.44	0.046	0.26	0.014
LeftHip flexion	13.87	0.064	0.43	0.010
LeftHip abduction	54.89	0.055	0.45	0.015
LeftHip rotation	44.03	0.098	0.69	0.015
LeftKnee flexion	22.77	0.080	0.44	0.016
LeftKnee abduction	47.87	0.043	0.41	0.012
LeftKnee rotation	58.09	0.061	0.46	0.016
LeftAnkle flexion	31.82	0.056	0.36	0.015
LeftAnkle abduction	7.29	0.081	0.24	0.015
LeftAnkle rotation	29.78	0.085	0.51	0.016
